# Cholera in Korea

**Published:** 1920-08-28

**Authors:** 


### Cholera in Korea.

We understand that an epidemic of cholera has broken out in Southern Korea. Up to the present 3,100 cases have been notified.

